# Understanding Barriers and Facilitators to Cardiovascular Rehabilitation Participation Among Rural Residents With Cardiovascular Disease in Brazil

**DOI:** 10.1111/phn.70072

**Published:** 2026-01-24

**Authors:** Mayara Moura Alves da Cruz, Lívia Tino de Roide, Anielle Brito de Oliveira Manzatto, Luiz Carlos Marques Vanderlei, José Vitor Barbosa Boro, Marcia Rodrigues Franco, Lais Manata Vanzella, Gabriela Lima de Melo Ghisi

**Affiliations:** ^1^ Department of Physiotherapy University Center of Adamantina (FAI) Adamantina Brazil; ^2^ Department of Physiotherapy School of Technology and Sciences São Paulo State University (UNESP) Presidente Prudente Brazil; ^3^ Department of Physiotherapy Centro Universitario UNA Contagem Brazil; ^4^ KITE Research Institute University Health Network Toronto Canada; ^5^ Department of Physical Therapy, Temerty Faculty of Medicine University of Toronto Toronto Canada

**Keywords:** cardiac rehabilitation, healthcare access, qualitative research, treatment adherence

## Abstract

**Objective:**

This study explored barriers to cardiovascular rehabilitation (CR) participation among individuals with cardiovascular disease (CVD) and risk factors in a rural, low‐resource setting.

**Design:**

A qualitative study using focus groups.

**Sample:**

21 individuals (76% women, 71% with CVD risk factors, 48% with incomplete elementary education) were recruited from a public health system.

**Measurements:**

Data were collected through six focus groups and analyzed using thematic analysis.

**Intervention:**

None.

**Results:**

Six overarching themes emerged. Participants had limited awareness of CR, with lack of knowledge being the primary barrier. Technological challenges hindered engagement with telerehabilitation programs. Medication was the most common treatment strategy, but patients expressed a need for more information. While physical exercise was recognized as important, barriers such as lack of time and motivation prevented participation. Perceptions of health status varied, with some reporting improvements and others experiencing deterioration. Family support and access to virtual programs were identified as facilitators of CR participation.

**Conclusions:**

Strategies to improve CR awareness, enhance virtual access, and strengthen patient education are needed to increase participation in rural populations.

## 1 Background

Cardiovascular disease (CVD) is the leading cause of mortality worldwide, accounting for approximately 18 million deaths annually (Martin et al., 2025). More than 80% of these deaths occur in low‐ and middle‐income countries, highlighting the disproportionate burden faced by resource‐limited settings (Roth et al., 2017). In Brazil, a middle‐income country, the estimated prevalence of CVD is 6,036 cases per 100,000 inhabitants (Institute for Health Metrics and Evaluation [IHME], 2016). Beyond its impact on mortality, CVD profoundly affects individuals’ physical, economic, and psychological well‐being. Extensive evidence corroborates the notion that those living with CVD often experience a notable decline in functional capacity and exercise tolerance, coupled with heightened occurrences of dyspnea, fatigue, angina, anxiety, and depression (Calegari et al., 2017; Nóbrega et al., 2013; Siqueira et al., 2017). CVDs also impose a significant burden on healthcare systems, resulting in considerable economic costs (Siqueira et al., 2017).

In this context, cardiovascular rehabilitation (CR) represents a key strategy for both primary and secondary prevention of CVD (Grace et al., 2016). CR is a comprehensive intervention that includes medical assessment, structured exercise training, risk factor management, patient education, and counseling (Brown et al., 2024). Robust evidence demonstrates that CR improves cardiorespiratory fitness and reduces coronary risk factors (Dibben et al., 2021; Franklin et al., 2020). In particular, the exercise component plays a central role in improving cardiovascular function, aerobic capacity, and quality of life, and is associated with reductions in mortality of up to 30% among cardiac patients (Calegari et al., 2017; Dibben et al., 2021; Franklin et al., 2020; Kabboul et al., 2018).

Despite its proven benefits, adherence to CR programs remains low (Grace et al., 2021). Only about 25% of individuals diagnosed with CVD who are eligible for CR actually enroll and start the program, with half of them ultimately withdrawing (Pollack et al., 2025). Similarly, approximately 50% of individuals with cardiovascular risk factors eligible for CR also prematurely withdraw (Forhan et al., 2013). Evidence from low‐ and middle‐income countries similarly demonstrates consistently low CR availability, referral, and attendance, with lack of physician referral emerging as the most frequently reported barrier to participation (Forhan et al., 2013).

Barriers to participation in CR have been extensively examined in high‐income countries at the healthcare system, provider, and patient levels (Iyngkaran et al., 2024). However, these barriers remain less well understood in middle‐income countries such as Brazil, where most CR programs are delivered through the public healthcare system (Britto et al., 2020). Existing Brazilian studies have largely relied on quantitative designs and focused on urban populations, offering limited insight into patient experiences and overlooking barriers faced by individuals living in rural areas (Borges et al., 2022; Ghisi et al., 2013; Santos et al., 2017; Sérvio et al., 2019).

Qualitative research is therefore needed to capture the contextual, experiential, and motivational factors that shape engagement and adherence to CR (Hammarberg et al., 2016). By exploring patients’ perspectives on CVD, treatment, and rehabilitation within rural settings, such approaches can inform more responsive and equitable CR models. This understanding is essential for supporting CR leaders and policymakers in Brazil to develop targeted strategies that improve access and participation among rural, minority, and underserved communities.

### 1.1 Research Question

What barriers and facilitators influence participation in cardiovascular rehabilitation among individuals with cardiovascular disease and cardiovascular risk factors living in a rural area of Brazil?

## 2 Methods

### 2.1 Design and Sample

This study employed a qualitative descriptive design using focus group (FG) discussions, with an inductive analytic approach to explore the barriers and facilitators hindering participation in CR programs among CVD patients living in a rural area in Brazil. Additionally, it seeks to ascertain whether these patients have received medical referrals to undergo CR. Study approval was obtained from the local Research Ethics Board (CAAE No. 59099722.6.0000.5496), and by the municipal health department. Data were collected between August and November 2022.

The participants in this study were individuals eligible for CR who had not previously engaged in CR programs. They were patients receiving medical support from the Unified Health System (SUS) at a basic healthcare unit in the city of Adamantina, state of São Paulo, Brazil. Adamantina has a population of 35,111 (2020 est.) and is classified as a rural area based on established criteria by the Office of Management and Budget (Health Resources and Services Administration, 2025). Specifically, it falls within the category of a micro area, defined as having an urban core population between 10,000 and 49,999 people. Additionally, Adamantina does not meet the threshold for a metropolitan area, which requires an urban core of at least 50,000 people.

Patients were recruited through telephone calls, during which the research team discussed details about the project. Those interested were then scheduled for an in‐person FG session. Prior to the start of the FG sessions, participants were given ample time to review the informed consent form and pose any questions. Only those who signed the informed consent form participated in the FG sessions.

A convenient sample of patients eligible for CR was invited to participate in the study. This sample included individuals diagnosed with CVD and/or presenting risk factors such as diabetes, hypertension, dyslipidemia, and/or obesity, who had never previously engaged in a CR program. Participants were required to be over 18 years old, regardless of gender/sex. The exclusion criteria included individuals with cognitive impairments, determined by assessing patients' orientation in time and space (Dsouza et al., 2021).

### 2.2 Measures

During the study visit, an initial evaluation was conducted to characterize the sample. Information collected included age, sex, medical history, medications, educational level, occupational status, referral to CR, and date of referral (if applicable). Following the initial assessment, face‐to‐face FG sessions were scheduled.

Six FG sessions were conducted, each comprising three to five participants and lasting approximately 45 min. The sessions were led by Dr. M.M.A.C., a female physiotherapy professor with a PhD and prior experience in qualitative research and CR, who had no previous relationship with the participants. A second researcher (L.T.R.) attended the sessions to take observational notes.

The FG sessions were guided by a semi‐structured interview script developed by experts in the field (Table 1). Each main question included probing questions to facilitate in‐depth discussion, and the moderator was able to introduce additional questions as needed to explore emerging topics.

**TABLE 1:** Focus group script.

1. Have you heard about cardiovascular rehabilitation? Where did you hear about it (i.e., hospital, medical appointment)? Who explained cardiac rehabilitation to you? What do you know about it?
2. What are the main reasons why you never participated in cardiovascular rehabilitation? Do you exercise alone? What type of exercise do you do?
3. How do you feel about your health? Do you feel that your health has deteriorated since your CVD diagnosis? Do you feel it has improved?
4. Do you believe that physical exercise is important for health? Why is exercise important? Do you know about the recommended way to perform physical exercise? (time, intensity and duration)
5. Would you feel safe exercising even if you have heart disease? What makes you feel safe about exercising? Do you feel safe exercising without supervision?
6. What do you think about the treatment you are currently undergoing for your heart disease? What do you know about the treatment you perform? What are the benefits?
7. What would facilitate your participation in a cardiovascular rehabilitation program? Do you have family support? What is your family support like?
8. Some cardiac rehabilitation programs are offered virtually (e.g., by telephone or videoconference). Would you be interested in this type of rehabilitation? What barriers do you think you would encounter to participate in a virtual program? (Ex.: technology, internet access, equipment, motivation). Do you have someone who could help you with the use of technology?
9. Is there anything that you would like to have been different in relation to the information you received for your care after the diagnosis of the disease? Is there anything else you would like to know about your diagnosis? Do you think you received all the important information? If not, what information would you like to have received?
10. Is there anything else you would like to say?

The FG sessions took place in a private room at the basic healthcare unit and were audio‐recorded using a portable digital recorder (OLYMPUS/VN‐8100 PC, Tokyo, Japan). All recordings were transcribed verbatim. At the end of each FG session, the researcher responsible for note‐taking summarized the main points discussed and invited participants to confirm or clarify the information to ensure accurate representation of their perspectives.

### 2.3 Analytic Strategy

Descriptive statistics were used to characterize the sample and are presented as means and standard deviations or as absolute numbers and percentages, as appropriate.

Qualitative data were analyzed using inductive, reflexive thematic analysis, following the six‐phase, iterative, and reflexive approach proposed by Braun and Clarke (2006). First, all focus group recordings were transcribed verbatim, and the research team immersed themselves in the data through repeated readings to achieve familiarization and gain an overall understanding of participants’ experiences (Phase 1). In Phase 2, initial codes were generated inductively from the data. Coding was conducted manually by two researchers, who independently identified meaningful segments of text relevant to barriers and facilitators of CR participation. Codes were compared and discussed to ensure consistency and to resolve discrepancies through consensus. During Phase 3, related codes were grouped to identify preliminary themes, capturing patterned meanings across the six focus group discussions. In Phase 4, themes were reviewed and refined by examining their coherence, internal consistency, and distinctiveness, both in relation to the coded extracts and the entire dataset.

Phase 5 involved defining and naming the final themes, ensuring that each theme clearly represented its core concept and contributed meaningfully to understanding barriers and facilitators to CR participation. Finally, in Phase 6, the themes were integrated into a coherent narrative and illustrated using representative participant quotations to support the analytic interpretations.

Data collection and analysis occurred concurrently, allowing emerging insights to inform subsequent focus group discussions. Thematic saturation was considered achieved when no new themes or substantive insights emerged across the six focus group discussions (Saunders et al., 2018). To enhance credibility, a summary of key discussion points was presented to participants at the end of each focus group for confirmation and clarification.

Rigor and trustworthiness were ensured through multiple strategies (Johnson et al., 2020). Credibility was supported by verbatim transcription of all focus group discussions, prolonged engagement with the data, and the use of participant quotations to illustrate each theme. Dependability and confirmability were enhanced through a systematic thematic analysis following Braun and Clarke's framework (Braun and Clarke, 2006), with independent coding by two researchers and consensus discussions during theme development.

## 3 Results

A total of 50 people were invited to participate in this study via telephone call. Of these, 21 individuals accepted the invitation, while 24 declined participation. Reasons for refusal included lack of transportation (*n* = 12), family commitment (*n* = 7), and lack of interest (*n* = 5). Additionally, five individuals did not answer the calls. The characteristics of individuals included in this study are detailed in Table 2.

**TABLE 2:** Participants’ characteristics (*N*=21).

Variables	100% (*N* = 21)
Age (years)	63.8 ± 10.8
BMI (kg/m^2^)	31.3 ± 7.0
Sex *(%, n)*	
Men	23.8% (*n*=5)
Women	76.2% (*n*=16)
Time of diagnosis (years)	12.0 ± 10.6
Clinical diagnosis *(%, n)*	
Cardiac arrhythmia	14.3% (*n* = 3)
Coronary insufficiency	4.8% (*n* = 1)
Heart failure	4.8% (*n*=1)
Acute myocardial infarction	4.8% (*n*=1)
Arterial hypertension	33.3% (*n*=7)
Diabetes mellitus	14.3% (*n*=3)
Obesity	14.3% (*n*=3)
Dyslipidemia	9.5% (*n*=2)
Referred to CR *(%, n)*	
Yes	4.8% (*n *= 1)
No	95.2% (*n *= 20)
Symptoms *(%, n)*	
Dyspnea	9.5% (*n *= 2)
Ischemic pain	9.5% (*n* = 2)
Fatigue	28.6% (*n* = 6)
Edema	4.8% (*n* = 1)
Palpitation	9.5% (*n *= 2)
Dizziness	4.8% (*n *= 1)
Weakness	14.3% (*n *= 3)
None	19.0% (*n* = 4)
Level of education *(%, n)*	
Incomplete primary education	38.1% (*n*=8)
Complete primary education	9.5% (*n*=2)
Incomplete secondary education	4.8% (*n*=1)
Complete secondary education	38.1% (*n*=8)
Post secondary education	9.5% (*n*=2)
Socioeconomic level *(%, n)*	
Up to 1 minimum wage	28.6% (*n*=6)
1‐2 minimum wage	23.8% (*n*=5)
2‐3 minimum wage	28.6% (*n*=6)
3‐5 minimum wage	9.5% (*n*=2)
5‐10 minimum wage	9.5% (*n*=2)

*Mean*±standard deviation. Legend: % = percentage; n = number of participants; BMI: body mass index; kg: kilograms; m: meters; CVD: cardiovascular diseases; CR: cardiovascular rehabilitation. The results are expressed as mean, standard deviation or in percentage and absolute number.

Six overarching themes were identified. Additional illustrative quotations supporting each theme are presented in Table 3.

**TABLE 3:** Additional participant quotations supporting identified themes.

Thematic category	Patient's quotes
*1‐ Lack of knowledge about CR and barriers to participation in the program*.	‐ *“I already heard it when I did physical therapy []” (Female, 54 years old, diagnosis of risk factors)* ‐ *“No one had ever told me, they just told me to take medicine, you know, I took such and such a medicine, and walked alone, but never, but I didn't even know it existed, you know? I didn't even know there was rehabilitation for this type of pathology disease.” (Female, 50 years old, diagnosis of risk factors)* ‐ *“Mine would also be the time, because there are days when I work, like today I'm not working and there are weeks when I work. There are weeks when I work the whole week and there are weeks when I don't, so for me it's confusing, it would be the time too, because it could only be after 6.” (Female, 44 years old, diagnosis of risk factors)* ‐ *“No, it's just that I take care of my mother, she is elderly and has to be always on top. Ah, it's just that after this pandemic, you… you always stay at home, stay at home, then settle down, actually I think that's it, it's comfort.” (Female, 57 years old, diagnosis of risk factors)*
*2‐ Barriers to cardiovascular telerehabilitation programs*.	‐ *“Me too, I have a cell phone, but I don't… I know how to use it, call, and answer it and, it's the essentials, those things that have everything, I don't know.” (Female, 68 years old, diagnosis of risk factors)*
*3 ‐ Medical advice upon receiving the diagnosis*.	‐ *“Yeah, you, taking a medicine without knowing what it is for, it's difficult, you take it, if you need it, but usually I, I learned to be curious, I like to do a little research, I look, but it's different of people asking.” (Female, 71 years old, diagnosis of CVD)* ‐ *“Ah, as soon as I went to the doctor, right. And now, you need to go for a walk because your cholesterol, your cholesterol is high. You go for a walk, you follow a diet, you explained what you should eat or not, but now my cholesterol is under control.” (Female, 59 years old, diagnosis of risk factors)* ‐ *“Yeah, I say it like this, the doctor diagnosed, followed up the pressure for 2 months, so it turned out, it really changed, if you will have to take the medicine, but the only thing he told me was to do some physical activity.” (Female, 69 years old, diagnosis of risk factors)* ‐ *“In the girls from… in psychology, and in… in nutrition I learned more things, you know, because the girls explain, talk about the foods that are harmful, which are not, so I thank you a lot, I learned a lot. But when you go to the… to the doctor like… then I think he doesn't have time, you know, to explain a lot of things […] if you go then, do what you're told, but you don't know where you're going arrive right.” (Female, 63 years old, diagnosis of risk factors)*
*4 ‐ Utilization of treatment strategies*.	‐ *“I only take medicine.” (Female, 57 years old, diagnosis of risk factors)* ‐ *“That's what I was going to say, because it might not cure us, but at least it slows it down, you live a little longer, right, because if we didn't have treatment, we wouldn't be able to bear it, because sometimes there are times when if you feel bad, and if you don't have the treatment, we can't stand it.” (Female, 63 years old, diagnosis of risk factors)*
*5 ‐ Barriers to exercise participation and perceived benefits*.	*‐ “Ah, I think that a trained person who knows how high we can go, I think we can go, yes, we get there alone, we are afraid […] then only a professional person who knows no, we don't. […] So we feel more insecure, you know, because then you're going to do it, you're already a little afraid, so I think it gives a little insecurity.” (Female, 63 years old, diagnosis of risk factors)* *‐ “Me, I didn't feel tired, I didn't feel tired when I walked like I do now, if I start walking now it takes away all the tiredness again, so for my tiredness it was fundamental, I improved a lot in terms of tiredness, which I was even starting to run for give more, right, then I started to relax, I started to relax for more than 3 or 4 months that I haven't walked anymore, so I saw that the tiredness came.” (Female, 69 years old, diagnosis of risk factors)*
*6 ‐ Perception of health status since diagnosis*.	‐ *“Ah, I feel good, apart from the tiredness, you know, I get really tired like this, then apart from the tiredness, the breathing is fine, the pressure, the diabetes is under control, the only problem is the circulation lately.” (Female, 44 years old, diagnosis of risk factors)* ‐ *"The heart started these days, I felt pain and burning, but the doctor said it's a lack of blood, I don't know." (Female, 63 years old, diagnosis of risk factors)* ‐ *“And then I thought it only got worse because you look back there and you see here in front you see that it got worse because we don't have the same disposition as in the past, right… It's a strange tiredness, it's not a tiredness that you worked on that sometimes you didn't even do anything, and you got out of bed already tired, you know, so it's very complicated, it's not easy.” (Female, 63 years old, diagnosis of risk factors)*

### 3.1 Theme 1: Lack of Knowledge About CR and Barriers to Participation in the Program

This theme reveals a significant knowledge gap regarding CR programs among participants. Only three individuals with CVD and three with cardiovascular risk factors had prior knowledge of CR, indicating a widespread lack of awareness. Even those who were aware could not articulate the benefits associated with CR or recall the source of their information. Three participants with cardiovascular risk factors had heard of CR, with their exposure typically occurring during regular physical activity sessions. The predominant barrier identified was the lack of knowledge about CR, affecting 15 participants. Additionally, other barriers included absence of medical referrals (mentioned by 20 participants), transportation difficulties (15 participants), lack of time (13 participants), family responsibilities (eight participants), and lack of interest (five participants).

“Yes, I know, vaguely, that there is, but I don't know how it is.” (Female, 71 years old, diagnosis of CVD)

“It's just that we live in Itamarati […] and it depends on the others to be able to leave the house, like now, my son brought us because we don't fear cars, there's nothing.” (Male, 77 years old, diagnosis of CVD)

“No, like, there are weeks like that, even UNIMED tells us to go there to exercise there, right, but I have my commitments, right? I have my leisure, I have a house by the river that maybe I'll say, this week I'm going to stay there, so I'm not going to come here in the city two, three times to do just that there, then I'm going to do my normal walk, right?” (Male, 67 years old, diagnosis of CVD)

### 3.2 Theme 2: Barriers to Cardiovascular Telerehabilitation Programs

This theme highlights the technological challenges faced by participants in engaging with telerehabilitation programs. Sixteen participants reported difficulties with technology, ranging from trouble navigating devices to unfamiliarity with necessary applications. These technological barriers significantly hindered their ability to participate in CR.

“Yeah, talk like that, I… I, my children, we talk on video like that, right? […] but I don't have a skill on the cell phone, if you understand, then there is an obstacle there that we are somewhat ignorant on something, right?physician” (Male, 67 years old, diagnosis of CVD)

“I have internet, but I don't know how to use it. I know how to take pictures, but I don't know how to send a message, I don't know how to open them, I don't know how to do anything, they're teaching me, my son, but I… my head isn't working yet.” (Female, 61 years old, diagnosis of risk factors)

### 3.3 Theme 3: Medical Advice Upon Receiving the Diagnosis

This theme provides insights into the medical guidance patients received at diagnosis. Most participants were prescribed medications and advised to integrate physical activity and healthy eating into their routines. However, these recommendations were often general, lacking clarity on the appropriate frequency, intensity, and duration of exercise. Additionally, participants were generally unaware of the main purposes and importance of their prescribed medications, leading them to seek additional information independently or from other healthcare professionals.

“Treatment? exercise. But I always did a lot of physical activity, always! So I walk very right? So, he said I should walk, those kinds of things like that, right? And take the medicine.” (Male, 67 years old, diagnosis of CVD)

“For me, I wrote a prescription, if you are going to take this one, look, 1 a day, 2 a day and such, there was no information.” (Male, 77 years old, diagnosis of CVD)

“Yes, exercising, going for a walk, it doesn't explain. You don't try to show us the benefit, you know. Go and do it, give the medicine and it's fine.” (Female, 71 years old, diagnosis of CVD)

### 3.4 Theme 4: Utilization of Treatment Strategies

This theme examines the treatment strategies adopted by participants. Medication use was the predominant treatment reported. Only one participant with CVD frequently engaged in walking, highlighting a reliance on medication over other treatment strategies such as regular physical activity.

“Yeah… the medicine was given, I'm taking it, there's no mistake.” (Male, 77 years old, diagnosis of CVD)

“The treatment in general is good, but it is not a treatment like that, how can I tell you, with a follow‐up so we know, give you strength to continue, you go to the SUS doctor, he gives you a medicine, you come back six months from now, sometimes you get better, that medicine didn't do you any good, you can't even complain that it didn't do any good, because we didn't pay anything, more generally speaking, in my case, for the heart it was working. ” (Female, 71 years old, diagnosis of CVD)

### 3.5 Theme 5: Barriers to Exercise Participation and Perceived Benefits

Despite recognizing the importance of exercise for managing their conditions, participants identified several barriers to engaging in physical activity. Lack of motivation (mentioned by 18 participants), apprehension about exercising without supervision (15 participants), and lack of time due to work commitments (six participants) were significant obstacles. However, participants acknowledged the psychosocial benefits of exercise, such as improved self‐esteem and better control of cardiovascular risk factors and symptoms.

“There has to be a person because alone we are relaxed, you know…” (Female, 67 years old, diagnosis of CVD)

“Yeah, it was like that. Before working with him, I was exercising every week with staff, which really encouraged me. But when my blood pressure got very high, the doctor asked me to stop and just walk alone. I stopped working with the staff, my mood changed, and I got discouraged. Now I feel I need to go back again.” (Male, 41 years old, diagnosis of risk factors)

“I'll be honest, I don't do it because I'm lazy, wasn't I supposed to be honest? I'm too lazy to leave the house and go for a walk, you know? I used to do it, every late Saturday and Sunday, I would leave here and go there near the forum and come back, it was a good walk, right? But then I started to get a little lazy.” (Female, 50 years old, diagnosis of risk factors)

### 3.6 Theme 6: Perception of Health Status Since Diagnosis

This theme explores participants' perceptions of their health status post‐diagnosis. Among patients with CVD, five reported deteriorated health characterized by increased fear, angina, and fatigue, while one reported improvement. Among those with cardiovascular risk factors, six reported maintaining their health status due to proper medication use, while four noted persistent symptoms like fatigue, edema, palpitations, weakness, and dizziness. The remaining 14 reported a decline in their health status, with fatigue being a predominant symptom impacting their daily routines.

“There are times when the heart hurts a lot, right? There's something, a pain as soon as I come up here, if I don't drink water I can't talk.” (Female, 79 years old, diagnosis of CVD)

“I'm vegetating, to tell you the truth, I get up in the morning, I manage to do something in the morning, then I do it until half past one. And really, in the afternoon I lie face up, lying down, because I can't stand to do anything anymore.” (Female, 71 years old, diagnosis of CVD)

“I feel good, I talk like that, I have a disposition like that which for my age is practically not normal, […] I have a lot of physical vigor like that, to, to walk, to run, to, I take a normal life, sometimes I even say that I don't even have a heart problem.” (Male, 67 years old, diagnosis of CVD)

## 4 Discussion

The findings of this study shed light on several crucial aspects surrounding CR participation among rural residents with cardiovascular disease in Brazil. A notable finding is the pervasive lack of awareness about CR programs, highlighting a significant knowledge gap that may impede access to essential CR services. Moreover, technological barriers emerged as a prominent obstacle, with a considerable number of participants encountering difficulties navigating telehealth platforms required for telerehabilitation programs. This underscores the importance of providing tailored support and guidance to enhance patients' comfort and proficiency with telehealth technologies. Furthermore, our study reveals insights into the medical advice received by patients upon receiving their diagnosis, indicating a need for more detailed and personalized guidance regarding exercise prescription and medication management. Additionally, our findings elucidate the barriers to exercise participation faced by patients, including time constraints, lack of motivation, and fear of unsupervised exercise. Importantly, despite these challenges, participants recognized the psychosocial benefits of physical activity, highlighting the need for interventions to address barriers and promote exercise adherence. Finally, patients' perceptions of their health status post‐diagnosis revealed a spectrum of experiences ranging from deterioration to improvement, with implications for treatment strategies and patient support.

### 4.1 Theme 1: Lack of Knowledge About CR and Barriers to Participation

The most prominent barrier identified was the lack of awareness regarding the existence and purpose of CR programs. Only a small number of participants—both with CVD and with cardiovascular risk factors—reported any prior knowledge of CR, and even among these individuals, understanding of its benefits was vague. This finding aligns with previous Brazilian studies demonstrating that insufficient patient knowledge is a major determinant of non‐participation in CR, particularly within the public health system (Ghisi et al., 2013; Ghisi et al., 2020; Nesello et al., 2015).

Limited awareness may reflect inadequate communication between healthcare professionals and patients at the time of diagnosis or follow‐up (Vermeir et al., 2015). Patients’ inability to recall receiving information about CR underscores missed opportunities for early education. Improving systematic patient education—through clear explanations at diagnosis, written materials, and reinforcement during routine visits—represents a critical strategy to overcome this barrier (Sudore and Schillinger, 2009). Evidence suggests that early and explicit discussions about CR benefits significantly increase participation and adherence, particularly among underserved populations (Ghisi et al., 2013; Ghisi, 2025).

### 4.2 Theme 2: Barriers to Cardiovascular Telerehabilitation Programs

Telerehabilitation has been proposed as a promising strategy to address geographic and structural barriers to CR access, especially in rural settings (Brouwers et al., 2024; Buyting et al., 2021). However, participants in this study reported substantial technological challenges, including limited digital literacy and difficulty navigating devices and applications. These findings echo prior research showing that technological barriers may disproportionately affect older adults and individuals with lower educational attainment (Franzoni and Motta, 2022; Gajarawala and Pelkowski, 2021; Md Fadzil et al., 2022).

To enhance the feasibility of telerehabilitation, preparatory interventions are needed. These may include hands‐on training sessions, simplified platforms, and the use of familiar technologies such as mobile phones rather than complex applications (Md Fadzil et al., 2022; Ouendi et al., 2025). Health professionals play a key role in building patient confidence and competence with technology before initiating remote CR. Ensuring patient readiness and comfort is essential, as acceptance and usability strongly influence the success of telerehabilitation program (Stark et al., 2023).

### 4.3 Theme 3: Medical Advice Upon Receiving the Diagnosis

Participants consistently described receiving general medical advice at diagnosis, primarily focused on medication use and broad recommendations for physical activity and diet. However, this guidance often lacked specificity regarding exercise intensity, frequency, and duration, and many patients reported limited understanding of their prescribed medications.

This finding highlights a gap between clinical recommendations and patient comprehension, reinforcing the importance of structured education as a core component of CR. CR guidelines emphasize the role of allied healthcare professionals (e.g., physiotherapists and physical education professionals) in risk stratification and exercise prescription (Grace et al., 2016). Strengthening interdisciplinary collaboration and ensuring that patients receive clear, tailored explanations may improve adherence and safety, particularly for individuals who feel uncertain or fearful about engaging in physical activity (Ghisi, 2025).

### 4.4 Theme 4: Utilization of Treatment Strategies

Medication emerged as the predominant treatment strategy among participants, with limited engagement in non‐pharmacological approaches such as regular physical activity. While medications were perceived as effective, several participants expressed a desire for more information about alternative or complementary strategies. This reliance on medication reflects a biomedical model of care and underscores the need to promote a more comprehensive approach to cardiovascular management (Leopold and Loscalzo, 2018). Patient education programs embedded within CR have been shown to improve knowledge, self‐management skills, and lifestyle behaviors (Shi et al., 2023). Expanding access to such educational interventions may help patients better understand the role of exercise, diet, and behavioral change alongside pharmacological treatment.

### 4.5 Theme 5: Barriers to Exercise Participation and Perceived Benefits

Despite recognizing the benefits of physical activity, participants identified multiple barriers to exercise participation, including lack of motivation, fatigue, time constraints, and fear of exercising without supervision. These barriers are well documented in the literature and are particularly salient among individuals with chronic conditions (Ghisi et al., 2013; Iyngkaran et al., 2024; Ragupathi et al., 2017; Sérvio et al., 2019).

Participants’ narratives highlighted the importance of supervision, encouragement, and social support in sustaining exercise behaviors. CR models that incorporate supervised, hybrid, or community‐based exercise programs may be especially beneficial in rural contexts (Karmali et al., 2014). Gradual progression, personalized exercise prescriptions, and psychosocial support can help address fear, improve motivation, and foster long‐term adherence (Bush et al., 2023).

### 4.6 Theme 6: Perception of Health Status Since Diagnosis

Participants’ perceptions of their health status since diagnosis varied widely, ranging from deterioration marked by fatigue and fear to perceived improvement and maintenance of function. These perceptions influenced motivation and engagement with treatment strategies, including exercise and lifestyle change. Understanding how patients perceive their health trajectory is critical for tailoring interventions (Koenders et al., 2025). Individuals reporting deterioration may require additional reassurance, symptom management, and support, whereas those perceiving stability or improvement may benefit from reinforcement strategies to sustain healthy behaviors (Ede et al., 2024). Incorporating patient‐reported outcomes and experiences into CR planning may enhance responsiveness to individual needs (Lavallee et al., 2016).

### 4.7 Implications for Practice and Policy

Taken together, these findings highlight the need for multifaceted strategies to improve CR participation in rural Brazil. Enhancing patient and provider education, strengthening referral pathways, expanding CR infrastructure, and tailoring telerehabilitation to patients’ technological capabilities are essential steps. Although Brazil's Unified Health System (SUS) offers universal coverage, disparities persist between urban and rural areas, particularly in access to specialized cardiovascular services. Investment in public CR programs, professional training, and community‐based models may help reduce these inequities. Addressing both structural and individual‐level barriers is crucial to improving CR utilization and ultimately reducing the burden of cardiovascular disease in low‐resource settings.

### 4.8 Limitations and Strengths

Caution is warranted while interpreting our findings. First, the generalizability of the results is limited by the fact that this study conducted exclusively with patients who attend the public health system. The study sample size was determined by data saturation, indicating that no new information emerged during the sixth FG session, resulting in 21 patients included. While this study adhered to the recommendations outlined in the literature (i.e., at least three FG sessions are typically needed to reach saturation, and the sample size should range from 5 to 50 participants) (Dworkin, 2012; Guest et al., 2017), we acknowledge that patients who utilize the public health care system may have a different socioeconomic status compared to those who use the private health care system. Therefore, additional studies should be conducted to investigate barriers to participation in CR faced by individuals utilizing the private health care system.

A key strength of this study lies in its qualitative and context‐specific approach. Previous research on barriers to CR participation in Brazil has largely relied on quantitative methods and focused on urban populations. In contrast, this study provides in‐depth qualitative insights into patient experiences in a rural setting, capturing perspectives that are often underrepresented in the literature. By focusing on a small rural municipality, this study offers valuable evidence to inform the development of contextually appropriate strategies to improve CR access for rural and underserved communities. Future research should build on these findings to further explore barriers across diverse care settings and to address socioeconomic disparities in CR participation in Brazil.

In conclusion, our study underscores that in low‐resource settings, the primary barrier hindering patients from participating in CR is the lack of awareness about the existence of such programs, potentially exacerbated by the scarcity of medical referrals. This emphasizes the importance of comprehensive planning and effective dissemination strategies targeting both the population and healthcare professionals to enhance treatment adherence. Additionally, there is a critical need for investment in public policies aimed at facilitating access and increasing referrals to CR centers, given the well‐established benefits of this intervention. By addressing these barriers and implementing targeted interventions, we can work toward improving the utilization of CR programs and ultimately enhancing cardiovascular health outcomes in low‐resource settings.

## Funding

This research did not receive any specific grant from funding agencies in the public, commercial, or not‐for‐profit sectors.

## Ethics Statement

All study procedures were approved by the University Center of Adamantina Ethics Committee (CAAE number: 59099722.6.0000.5496) and adhered to the Declaration of Helsinki. Informed consent was obtained from all participants.

## Conflicts of Interest

The authors declare no conflict of interest.

## Data Availability

The data that support the findings of this study are available on request from the corresponding author. The data are not publicly available due to privacy or ethical restrictions.
